# Epigenetic Regulation of Tumor Suppressors by *Helicobacter pylori* Enhances EBV-Induced Proliferation of Gastric Epithelial Cells

**DOI:** 10.1128/mBio.00649-18

**Published:** 2018-04-24

**Authors:** Saurabh Pandey, Hem Chandra Jha, Sanket Kumar Shukla, Meghan K. Shirley, Erle S. Robertson

**Affiliations:** a Departments of Otorhinolaryngology-Head and Neck Surgery, and Microbiology, the Tumor Virology Program, Abramson Cancer Center, Perelman School of Medicine at the University of Pennsylvania, Philadelphia, Pennsylvania, USA; University of Pittsburgh School of Medicine

**Keywords:** Epstein-Barr virus, *Helicobacter pylori*, epigenetics, transformation

## Abstract

Helicobacter pylori and Epstein-Barr virus (EBV) are two well-known contributors to cancer and can establish lifelong persistent infection in the host. This leads to chronic inflammation, which also contributes to development of cancer. Association with H. pylori increases the risk of gastric carcinoma, and coexistence with EBV enhances proliferation of infected cells. Further, H. pylori-EBV coinfection causes chronic inflammation in pediatric patients. We have established an H. pylori-EBV coinfection model system using human gastric epithelial cells. We showed that H. pylori infection can increase the oncogenic phenotype of EBV-infected cells and that the cytotoxin-associated gene (CagA) protein encoded by H. pylori stimulated EBV-mediated cell proliferation in this coinfection model system. This led to increased expression of DNA methyl transferases (DNMTs), which reprogrammed cellular transcriptional profiles, including those of tumor suppressor genes (TSGs), through hypermethylation. These findings provide new insights into a molecular mechanism whereby cooperativity between two oncogenic agents leads to enhanced oncogenic activity of gastric cancer cells.

## IMPORTANCE

We have studied the cooperativity between H. pylori and EBV, two known oncogenic agents. This led to an enhanced oncogenic phenotype in gastric epithelial cells. We now demonstrate that EBV-driven epigenetic modifications are enhanced in the presence of H. pylori, more specifically, in the presence of its CagA secretory antigen. This results in increased proliferation of the infected gastric cells. Our findings now elucidate a molecular mechanism whereby expression of cellular DNA methyl transferases is induced influencing infection by EBV. Hypermethylation of the regulatory genomic regions of tumor suppressor genes results in their silencing. This drastically affects the expression of cell cycle, apoptosis, and DNA repair genes, which dysregulates their associated processes, and promotion of the oncogenic phenotype.

## INTRODUCTION

Helicobacter pylori functions together with Epstein-Barr virus (EBV) as a group 1 carcinogen which contributes to the development of gastric cancer (GC) (1, 2). EBV is associated with several types of lymphoid and epithelial cancers, including GC (3–5). Previous studies also suggested that EBV can transform primary gastric epithelial cells *in vitro* (6). Therefore, EBV is likely not a passive carrier but an active oncogenic virus contributing to early events in development of GC (4, 7). More importantly, EBV-positive GC harbors EBV DNA in a uniform, monoclonal presence in all carcinoma cells (7, 8), and patients show high antibody titers against EBV prior to diagnosis (9). In a study with pediatric patients, coinfection with H. pylori and EBV caused severe gastritis and chronic inflammation compared to individual infection of the respective pathogens (10). Further, many reports have highlighted the significance of EBV and H. pylori in GC. However, not much has been done to investigate the molecular mechanism linked to coinfection of these agents or the degree or extent of associated pathologies, in this case, those represented by gastric cancer.

Pathogens coexisting with host cells can act as effectors of gene regulation through epigenetic modifications. This impacts the overall etiology and pathogenesis associated with the respective disease. For example, the periodontal bacterium Porphyromonas gingivalis can induce EBV reactivation through chromatin modification (11). This bacterium-virus synergy affects EBV-associated periodontal pathology (11). Furthermore, H. pylori and EBV have also been shown to impact epigenetic modifications of host cells (12, 13). This relationship, which exists within the microbial milieu and modulates host gene expression, can directly impact disease pathology.

We investigated the molecular mechanism to elucidate the underlying strategy, which involves cooperation of H. pylori in EBV-driven proliferation of gastric epithelial cells. We developed an H. pylori and EBV coinfection model by the use of NCI-N87 human gastric epithelial cells, which is an excellent system mimicking human gastric epithelium (14). We demonstrated that the cytotoxin-associated gene (CagA) protein encoded by H. pylori promoted EBV-mediated proliferation of infected cells in our model system. Furthermore, we determined that the epigenetic status of EBV-infected cells was modulated as a consequence of H. pylori coinfection. This reprogramming resulted in upregulation of DNA methyl transferases (DNMTs), known epigenetic modifiers, leading to promoter methylation of CpG-rich islands of cell cycle-, DNA repair-, and apoptosis-related tumor suppressor genes (TSGs). Hypermethylation of the regulatory regions of these TSGs resulted in changes in cellular transcription profiles and the microenvironment that favored oncogenesis.

## RESULTS

### H. pylori enhances EBV infection and virion production in human gastric epithelial cells.

**FIG 1:**
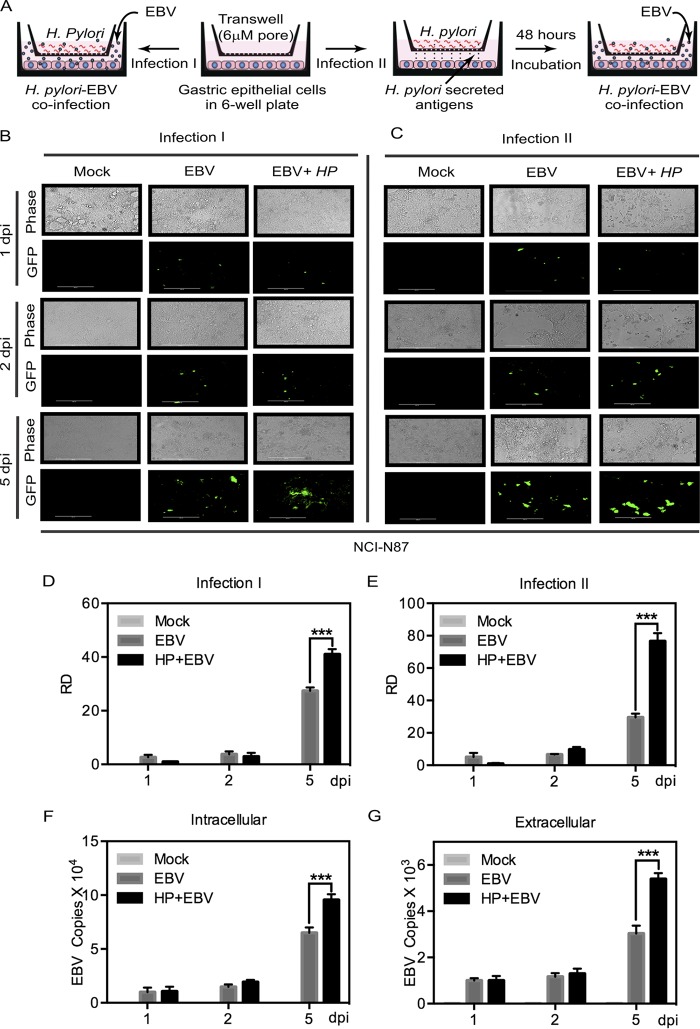
H. pylori increases the efficiency of EBV infection and virion production in gastric cells. (A) NCI-N87 cells were infected with GFP-EBV with and without coinfection with H. pylori. The infection scheme is depicted in the diagram. NCI-N87 cells were cultured in 6-well plates, and exposure to H. pylori was performed using a transwell filter. Secretory antigens cross the transwell to which NCI-N87 cells are exposed. EBV, being smaller than the membrane cutoff pore size, has access to the whole volume. (B) Infection I (simultaneous infection of EBV and H. pylori). (C) Infection II. H. pylori and EBV were infected in sequence, and preinfection of H. pylori was done for 48 h. Infection performed without H. pylori (i.e., infection using only EBV) and mock infection (i.e., no infection [mock]) were used as controls. The experiment was performed three times, and images from a representative experiment are presented. (D and E) The fluorescent micrograph results were quantified using densitometric analyses, and data from the (D) simultaneous infections and (E) sequential infections are presented as relative density (RD) values. (F and G) Relative EBV-GFP DNA copy numbers were quantified as a measure of EBV load using (F) intracellular and (G) extracellular released EBV in the culture and real-time qPCR.

The interplay between EBV and H. pylori and the downstream implications of that interplay were investigated by establishing an *in vitro* coinfection system using the NCI-N87 gastric cell line, which mimics cells of the gastric epithelium (14, 15). H. pylori and EBV cultures were established as described in Materials and Methods. As expected, H. pylori showed regular spiral morphology by Gram stain as described earlier (16) (see Fig. S1 in the supplemental material). We studied two possible approaches for construction of a system using EBV and H. pylori coinfection. The first approach was that of simultaneous infection, where H. pylori and EBV were incubated simultaneously with the cells (infection I), and the second was that of sequential infection, where the gastric epithelial cells were first incubated with H. pylori and then subjected to infection with EBV (infection II). The results showed that sequential infection of the culture with EBV following preincubation with H. pylori was the more effective approach, as it provided to the cells an opportunity to establish and maintain a microenvironment suitable for more-efficient viral infection (Fig. 1A). After infection, fluorescent microscopic imaging and densitometric quantitation were performed at specific time points prior to exposure of the cells with H. pylori. This method showed greater efficiency of infection than simultaneous and control infections as determined by examination of green fluorescent protein (GFP) signals (Fig. 1B and C). A significant increase in fluorescence at 5 days postinfection (dpi) as determined by densitometric analysis (Fig. 1D and E) was also observed. Thereafter, the infection II setup was used in all of the experiments that followed.

10.1128/mBio.00649-18.2 FIG S1  H. pylori shows spiral morphology. H. pylori was cultured as described in Materials and Methods and subjected to Gram staining prior to setup and infection. The usual spiral morphology is shown. Download FIG S1, TIF file, 1.1 MB. Copyright © 2018 Pandey et al. 2018 Pandey et al. This is an open-access article distributed under the terms of the Creative Commons Attribution 4.0 International license.

The relative viral copy numbers measured at 2 and 5 dpi for both cell-associated and extracellular virion particles were significantly and reproducibly higher during coinfection than during EBV infection alone (Fig. 1F and G). Similarly, relatively higher viral loads were observed when the cells were exposed to H. pylori culture supernatant, which would have contained secreted factors (Fig. S2). The level of relative density (RD) of the GFP signal clearly showed a strong increase in efficiency of viral infection by 5 to 7 dpi (Fig. S2A and B). Interestingly, the levels of intracellular and extracellular virions also increased in the cells exposed to H. pylori supernatant (Fig. S2C and D).

10.1128/mBio.00649-18.3 FIG S2  H. pylori culture medium enhances the potency of EBV infection and virion production in gastric cells. (A) NCI-N87 cells infected with GFP-EBV in the presence of cell-free H. pylori soup and brain heart infusion (BHI) media as a control were subjected to fluorescent microscopic imaging at different time points (1, 2, 5, and 7 dpi). (B) Relative fluorescent intensity levels were calculated and are presented. (C and D) DNA loads from (C) intracellular and (D) extracellular EBV were also enumerated. Download FIG S2, TIF file, 2.2 MB. Copyright © 2018 Pandey et al. 2018 Pandey et al. This is an open-access article distributed under the terms of the Creative Commons Attribution 4.0 International license.

**FIG 2:**
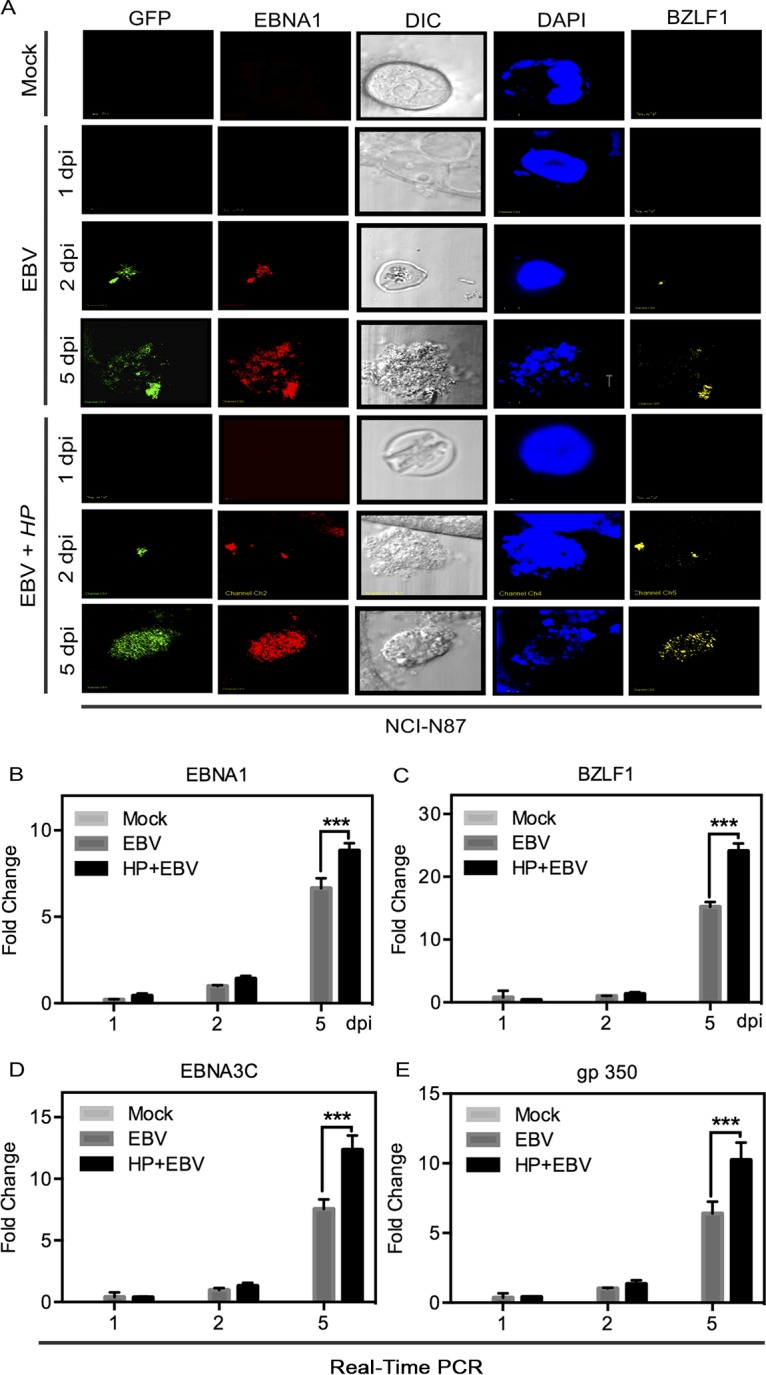
H. pylori-EBV coinfection results in upregulation of EBV latent and lytic genes. (A) NCI-N87 cells were coinfected with H. pylori-EBV-GFP along with the control. Cells were examined for the expression of latent and lytic marker genes (EBNA1 and BZLF1, respectively) at different time points (1, 2, and 5 dpi) using fluorescence microscopy. DIC, differential interference contrast; DAPI, 4′,6-diamidino-2-phenylindole. (B to E) The expression profiles of EBNA1 (B), BZLF1 (C), EBNA3C (D), and gp350 (E) were analyzed at the transcript level using RT-qPCR.

EBV can establish two distinct modes of postinfection replication, namely, latent replication and lytic replication. Furthermore, latent infection promotes cellular oncogenesis. However, recent studies showed that lytic activation is also a major contributor to the oncogenic process (17). To identify the mode of viral replication, we examined the expression status of two EBV genes: genes EBNA1 (latent infection) and BZLF1 (lytic infection) (Fig. 2). The results showed that the expression levels of both EBNA1 and BZLF1 were enhanced during coinfection compared to the results seen with infection with EBV alone as determined by immunofluorescence (Fig. 2A). The transcript levels of EBNA1 were also increased (Fig. 2B). Notably, we observed a significant increase in the levels of the immediate early transcript BZLF1 at 5 dpi, indicating a significant burst of lytic replication, early during infection. Moreover, when cells were exposed to H. pylori prior to EBV infection, the expression levels of BZLF1 increased from 15-fold to 24-fold, which represents a greater increase in viral lytic activity (Fig. 2C). These data validated our fluorescence data determined using the anti-BZLF1 monoclonal antibody (compare the panels at the right side in Fig. 2A and C). Similar findings were obtained when cells were preincubated with H. pylori culture supernatant (Fig. S3C). We found that the expression levels of the latent EBNA1 gene and the immediate early BZLF1 gene carried by EBV were upregulated as seen by immunofluorescence upon preincubation with H. pylori culture supernatant followed by EBV infection. Furthermore, an increase in transcript levels was also seen for the gp350 lytic gene (Fig. S3D). These results demonstrated that factors secreted by H. pylori are likely important in driving upregulation of these virus-carried genes.

10.1128/mBio.00649-18.4 FIG S3  Exposure of EBV-infected cells to H. pylori culture medium results in upregulation of EBV latent and lytic genes. (A) NCI-N87 cells were infected with EBV GFP in the presence of H. pylori supernatant. Cells were examined for the expression of EBV latent and lytic genes (EBNA3C and BZLF1, respectively) by the use of fluorescence microscopy at different time points. (B to D) The transcription profiles of latent gene EBNA1, lytic gene BZLF1, and GP350 viral glycoproteins were investigated using RT-qPCR at different time points (1, 2, 5, and 7 dpi). Download FIG S3, TIF file, 2.2 MB. Copyright © 2018 Pandey et al. 2018 Pandey et al. This is an open-access article distributed under the terms of the Creative Commons Attribution 4.0 International license.

### Exposure to H. pylori resulted in increased production of infectious virions in EBV-infected gastric epithelial cells.

**FIG 3:**
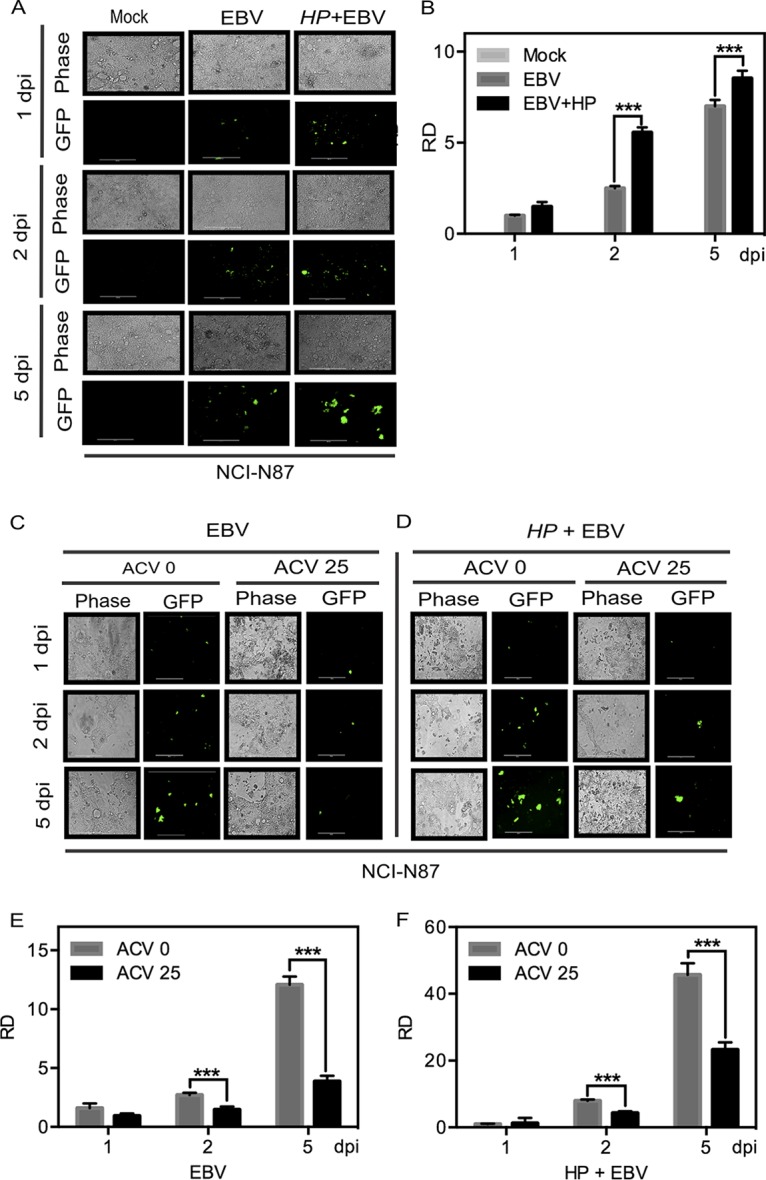
H. pylori-EBV coinfection results in an increase of viral infectivity and is controlled by the antiviral drug ACV. (A) Infectious virions produced in the first coinfection and in the control experiment were collected, and a fresh infection was set up. Then, levels of viral load and infectivity were estimated by the use of a microscope at different time points. Data from one representative experiment are representative of results from experiments performed in triplicate. (B) Fluorescent intensity was quantitated for the triplicate experiments and normalized against the mock infection data, and relative expression density data are plotted. (C and D) Microscopic analysis of GFP expression was performed in the absence (C) and presence (D) of H. pylori (*HP*) by comparing the effects of 25 µM ACV in both groups along with a no-ACV control. The experiment was performed in triplicate, and data from a representative experiment are presented. (E and F) Relative density (RD) data corresponding to GFP expression as a measure of EBV abundance in the absence (E) and presence (F) of H. pylori were plotted after quantitation of the fluorescent intensity measured in all three experiments.

To further support our results described above, which showed augmentation of lytic EBV infection when cells were exposed to H. pylori prior to EBV infection, we evaluated the viability of GFP-EBV progeny produced after infection. Progeny of GFP-EBV were collected, filtered, and used to infect a fresh culture of NCI-N87 gastric cells. After infection, the level of GFP expression in the newly infected cells was measured at 1, 2, and 5 dpi. As expected, the relative GFP density was observed to be higher when infection was performed using progeny EBV collected as described above from the supernatant obtained from coinfected NCI-N87 cells (Fig. 3A). Interestingly, the fluorescence intensity doubled after 2 dpi in the case of cells infected with supernatant from the cultures coinfected with H. pylori and EBV (Fig. 3A). Furthermore, the virus produced from the coinfected gastric cells showed greater lytic burst and, within 48 h postinfection, showed GFP signals that were more than two times higher in terms of relative density than those seen with the virions produced from gastric cells infected with EBV alone (Fig. 3B). Nevertheless, the changes in the levels of GFP signals seen at 5 dpi were not as dramatic, but the signals from the virions obtained from coinfected gastric epithelial cells were consistently higher (Fig. 3B). These results showed that the ability to infect new gastric cells was enhanced in EBV produced from gastric cells that had previously been exposed to H. pylori. Interestingly, similar observations were recorded when, instead of H. pylori bacteria, H. pylori supernatant or cell-free culture medium was used; the results showed enhanced levels of GFP signals, which indicated infection of NCI-N87 cells (Fig. S4A and B). To determine if the changes in the levels of the signals were due to passive GFP transfer, we incubated NCI-N87 cells as described above with supernatant obtained from GFP-transfected HEK-293 cells as a control. No passive transfer of GFP was seen in the NCI-N87 cells, indicating that the levels of GFP signals measured in the experiments described above were not due to passive transfer (Fig. S4C).

10.1128/mBio.00649-18.5 FIG S4  Exposure of EBV-infected cells to H. pylori culture medium results in an increase in infectious virion production in EBV-infected gastric cells (A) NCI-N87 cells were infected with EBV GFP in the presence of H. pylori soup. The infection virions produced in the first infection, along with those present in the control, were collected, and a fresh infection was set up in a similar way. Then, the viral load was measured using fluorescence microscopy at different time points. (B) Fluorescence micrograph results were quantitated, and relative fluorescent intensity data are presented. (C) Mock infection to test passive GFP uptake. To rule out any possibility of passive GFP uptake by dead cells or their debris, a control reaction was performed. Cells expressing GFP after transfection of plasmid pEGFP vector were subjected to similar procedures of virus purification, and the collected pellet was used to infect the NCI-N87 cells, in the presence and absence of H. pylori. The results indicated that the no appearance of fluorescence signals due to GFP/cell components. Download FIG S4, TIF file, 2.4 MB. Copyright © 2018 Pandey et al. 2018 Pandey et al. This is an open-access article distributed under the terms of the Creative Commons Attribution 4.0 International license.

### Acyclovir inhibits efficient production of EBV progeny from gastric epithelial cells coinfected with H. pylori.

In our studies described above, we found that the density of fluorescence was significantly higher in the coinfection model than in the model using EBV infection alone. Using acyclovir [ACV; 9-(2-hydroxyethoxy methyl) guanine], it was observed that there was an almost complete shutdown of signals indicating viral infection by 5 days in the infections performed with EBV alone (Fig. 3C and D). However, in coinfection experiments performed in the presence of the antiviral drug acyclovir, we observed a slightly higher level of signal which indicated that there was a low level of viral infection, although this signal was about 50% lower than that seen without the drug (Fig. 3; compare panels C and E with panels D and F). This efficiency indicated that coinfection of H. pylori and EBV increased the efficiency of infection by the virions produced even when replication was blocked by the inhibitor (Fig. 3D and F).

### CagA encoded by WT H. pylori is important for enhanced EBV infection of gastric epithelial cells.

**FIG 4:**
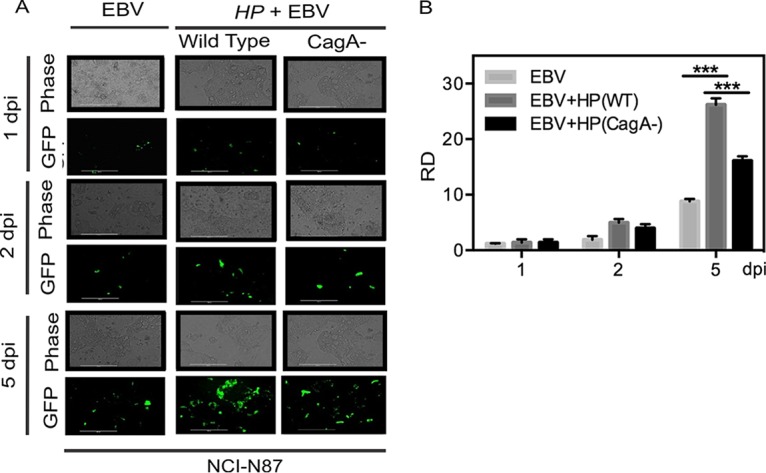
H. pylori-encoded CagA enhances the efficiency of EBV infection in gastric epithelial cells. (A) Fluorescence and phase-contrast images were captured at different time points in coinfection of EBV-GFP along with H. pylori wild-type and CagA mutant strains. The experiment was performed in triplicate, and data from a representative experiment are presented. (B) Relative fluorescence density data were plotted after quantification of the results from all three experiments.

Using the coinfection model described above, an H. pylori CagA-deleted mutant [(CagA^−^)] was evaluated for its ability to enhance EBV infection of gastric cells (18). The progeny EBV was quantified in comparison to the results seen with the isogenic wild-type (WT) H. pylori [H. pylori(WT)] strain. Notably, our results showed that there was a significant drop in efficiency of infection in the absence of the CagA gene (Fig. 4A). At 2 dpi, there was little or no difference observed between EBV coinfected with wild-type (WT) H. pylori and EBV coinfected with H. pylori(CagA^−^). At 5 dpi, however, there was an approximately 40% drop in signal (Fig. 4A). The quantitation of these results indicated that CagA is an important contributor to the increased production of EBV virions and to the potency of its infection in the context of coinfection with H. pylori (Fig. 4B). The dramatic drop in signal seen with the H. pylori CagA mutant compared to WT H. pylori at 5 dpi was, however, greater than that seen with EBV alone (Fig. 4B). Further studies performed using the supernatant of NCI-N87 cells after infection with EBV alone, coinfection with H. pylori(WT) and EBV, and coinfection with H. pylori(CagA^−^) and EBV to infect fresh gastric NCI-N87 cells showed a dramatic drop in infection potency at 2 dpi, with some recovery at 5 dpi (Fig. S5A and B). The GFP signal for infection was also clearly lower with the CagA mutant culture supernatant from infected NCI-N87 cells at 5 dpi, although less than the relative density of the signals at 2 dpi (Fig. S5B). Interestingly, the results seen after blocking early replication using acyclovir did not show any major difference in the levels of the resulting signals in comparisons of the results of H. pylori(WT) coinfection to the results of H. pylori(CagA^−^) coinfection of NCI-N87 cells with EBV. This indicated that the levels of replication of the virions in the two coinfections in the presence of acyclovir were inhibited to similar extents (Fig. S5; compare panels C and E with panels D and F).

10.1128/mBio.00649-18.6 FIG S5  The culture medium of H. pylori-encoded CagA is important for enhanced active virion production in gastric epithelial cells. (A) NCI-N87 cells were infected with GFP EBV in the presence of H. pylori wild-type and H. pylori CagA^−^ culture soup. Fluorescence microscopy was employed to analyze the viral load. (B) Fluorescent micrograph results were quantitated, and relative fluorescence density data are presented. (C and D) In the presence of an inhibitory concentration of ACV, the viral load was evaluated in coinfection with preexposure and in the absence of H. pylori CagA^−^ culture soup. (E and F) Fluorescent micrograph results were quantitated, and relative fluorescent intensity data are presented. Download FIG S5, TIF file, 3.5 MB. Copyright © 2018 Pandey et al. 2018 Pandey et al. This is an open-access article distributed under the terms of the Creative Commons Attribution 4.0 International license.

### H. pylori-EBV coinfection led to aberrant methylation of genes regulating major cellular processes.

**FIG 5:**
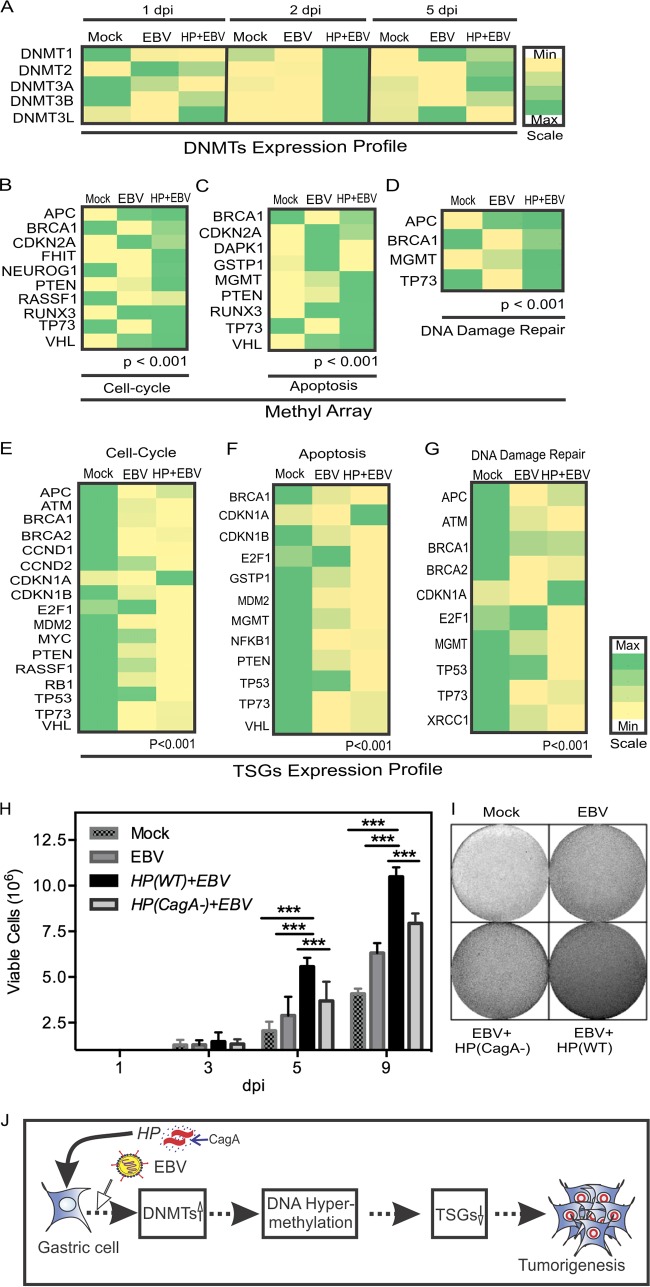
H. pylori and EBV coinfection alters the expression of host DNMTs, leading to hypermethylation of TSGs, downregulation of TSG expression, and proliferation of gastric cells. (A) Gene expression analysis of host DNA methyl transferases in response to EBV infection and H. pylori-EBV coinfection. After infection, cell samples were collected at different time points. qPCR analysis was performed, and relative transcriptional changes are presented in the form of a heat map. Expression of individual DNMTs was normalized to the results determined for a no-infection control. The experiment was performed three times. Min, minimum; Max, maximum. (B to D) Methyl array analysis for promoter methylation profiling of TSGs of cell cycle (B), apoptosis (C), and DNA damage repair (D) pathways in response to mock infection (Mock), EBV infection, and H. pylori–EBV coinfection in NCI-N87 cells was performed. Relative methylation data are presented as a heat map where individual genes are normalized against mock infection. In response to large-scale methylation of genomic DNA, the expression profiles of cell cycle, apoptosis, and DNA damage repair TSGs were modulated. The levels of modulation were measured through RT-qPCR. (E to G) Relative expression levels of cell cycle (E), apoptosis (F), and DNA damage repair (G) TSGs were plotted as a heat map normalized against the results of mock infection. Expression of these TSGs was mostly dampened in the case of coinfection. (H) Using the standard trypan blue exclusion assay method, proliferation of GC cells under different conditions was scored. (I) A colony formation assay was performed using a previously described method (68) and crystal violet staining. (J) A model depicting the modulation of the cell cycle, apoptosis, and DNA damage repair pathways that can lead to tumorigenesis of EBV-H. pylori-positive gastric epithelial cells. H. pylori-promoted augmentation of DNMTs in host cells changes hypermethylation at the genome level and represses tumor suppressor gene expression and therefore function. This results in an increase in the oncogenic activities of the infected cells.

In previous studies, we showed that EBV infection of primary B cells leading to transformation relied on dysregulation of the cellular methylation program that resulted from hijacking major cellular epigenetic modifiers (19, 20). DNMTs regulate cellular epigenetic methylation patterns, including promoter hypermethylation of TSGs and oncogenic hypomethylation, in EBV-associated cancers (21, 22). However, the process by which H. pylori infection influences EBV-associated GC through an epigenetics-dependent molecular mechanism has not been previously elucidated. In this study, we investigated the relative levels of expression of DNMTs using the coinfection model system at 1, 2, and 5 dpi using NCI-N87 gastric cells, which mimic primary gastric epithelium cells. Upon coinfection with H. pylori and EBV, the NCI-N87 cells were highly methylated at 2 dpi, with a significant increase in expression of all DNMTs examined, and the DNMTs remained induced, except for the results seen with DNMT3L, which showed little or no detection (Fig. 5A). Further, we investigated the promoter methylation patterns of a number of TSGs (using EpiTect methyl II PCR arrays, without the need for bisulfite sequencing [SABiosciences, Frederick, MD] of the regulatory regions of the human tumor suppressor genes), as methylation impacts the oncogenic potential of the infected NCI-N87 cells. Our findings strongly demonstrated that EBV infection of NCI-N87 induced promoter methylation of a number of TSGs and that the presence of H. pylori further induced the level of methylation of these TSGs, which are involved in cell cycle, apoptosis, and DNA damage responses (Fig. 5B, C, and D). The hypermethylated TSGs included APC (adenomatous polyposis coli), BRCA1, FHIT, MGMT (methyl guanine DNA methyl transferase), NEUROG1, PTEN (phosphatase and tensin homologue), RUNX3, TP73, and VHL genes (Fig. 5B, C, and D). TSGs showing no change in status or decreased methylation in comparisons between the results seen with EBV infection alone and coinfection with EBV and H. pylori included the CDKN2A, DAPK1, GSTP1, and RASSF1 genes (Fig. 5B, C, and D). A detailed description of the genes affected by methylation reprogramming is provided in Table S1 in the supplemental material. The complete list of 22 genes and the associated functions can be found in Table S2.

10.1128/mBio.00649-18.8 TABLE S1  List of TSGs investigated in the methylation profiling experiment. Download TABLE S1, DOCX file, 0.1 MB. Copyright © 2018 Pandey et al. 2018 Pandey et al. This is an open-access article distributed under the terms of the Creative Commons Attribution 4.0 International license.

10.1128/mBio.00649-18.9 TABLE S2  Categories of TSGs in the methyl profiling experiment. Download TABLE S2, DOCX file, 0.1 MB. Copyright © 2018 Pandey et al. 2018 Pandey et al. This is an open-access article distributed under the terms of the Creative Commons Attribution 4.0 International license.

We clustered the TSGs into distinct functional classes, and, from that analysis, we showed that *in vitro* infection of the NCI-N87 gastric cells by H. pylori and EBV was associated with transcriptional repression of a number of cell cycle genes (APC, BRCA1, CDKN2A, FHIT, NEUROG1, PTEN, RUNX3, TP73, and VHL), genes involved in apoptosis (BRCA1, CDKN2A, MGMT, PTEN, RUNX3, TP73, and VHL), and genes involved in DNA damage repair (APC, BRCA1, MGMT, and TP73) (Fig. 5B, C, and D) (Table S2).

### Coinfection of gastric NCI-N87 cells with H. pylori and EBV led to transcription repression of TSGs through methylation of their promoter regulatory elements.

Exposure to carcinogens can change the methylation patterns of regulatory genes and results in suppression of their expression (23). To support our findings indicating that expression of DNMTs is significantly induced during coinfection of NCI-N87 gastric epithelial cells with H. pylori and EBV, we examined cellular genes in major functional classes (cell cycle, apoptosis, and DNA damage repair) at the transcript level. We performed quantitative PCR (qPCR) using experimental settings similar to those previously described (20). A detailed description of the genes examined, which were regulated through transcriptional reprogramming, is provided in Table S3.

10.1128/mBio.00649-18.10 TABLE S3  List of categories of TSGs regulated by transcription reprogramming. Download TABLE S3, DOCX file, 0.05 MB. Copyright © 2018 Pandey et al. 2018 Pandey et al. This is an open-access article distributed under the terms of the Creative Commons Attribution 4.0 International license.

The results showed that coinfection with H. pylori and EBV was associated with deregulation of cell cycle, apoptosis, and DNA damage repair genes (Fig. 5E, F, and G). The majority of the genes investigated showed a reduction in transcript levels, except for CDKN1 (p21), which showed increased levels, and the results are represented as a heat map (Fig. 5E, F, and G). The TP73, VHL, APC, BRCA1, and BRCA2 genes were downregulated, but the changes were not as dramatic, suggesting that a stringent balance is required for their expression in NCI-N87 gastric cells coinfected with H. pylori and EBV (Fig. 5E, F, and G). Further analysis of the associated cellular pathways using Ingenuity pathway analysis supported our findings indicating that dysregulation of the TSGs is likely critical for development of gastric cancer mediated by H. pylori and EBV, as the majority of the genes that were dysregulated were connected to gastric cancer development (Fig. S6A).

10.1128/mBio.00649-18.7 FIG S6  (A) Dysregulation of tumor suppressors has a significant potential for gastric cancer development. Associations of host tumor suppressors with gastric cancer, affected by H. pylori and EBV coinfection, were analyzed using an Ingenuity pathway analysis (IPA) program that showed a highly significant *P* value of 3.27E−18 for such association. (B) H. pylori exposure alone has a negative impact on the proliferation of gastric cells. Proliferation of gastric cells was measured upon H. pylori wild-type and CagA^−^ mutant exposure. (C) Relative gene expression profiles of NCI-N87 cells upon H. pylori wild-type and CagA^−^ mutant exposure. Relative expression profiles of many host TSGs were evaluated upon exposure to H. pylori (WT) alone. Data were analyzed, and no statistically significant changes were observed after H. pylori treatment. Download FIG S6, TIF file, 2.2 MB. Copyright © 2018 Pandey et al. 2018 Pandey et al. This is an open-access article distributed under the terms of the Creative Commons Attribution 4.0 International license.

Gastric epithelial NCI-N87 cells were further exposed to H. pylori and EBV (along with controls) to investigate their viability and proliferative activities (Fig. 5H and I). We found that coinfection enhanced the viability of the cells as seen by trypan blue exclusion assay and increased colony formation, as would be expected for a more robust cancer phenotype (Fig. 5H and I). A model representing coinfection of the gastric mucosa and the sequence of events resulting in tumorigenesis is provided (Fig. 5J). The H. pylori(CagA^−^) mutant showed increases similar to those seen with infection with EBV alone (Fig. 5H and I). Importantly, the results showed that infections performed with H. pylori(WT) and H. pylori(CagA^−^) without EBV did not have any direct effect on the oncogenic phenotype or on the transcript levels of the TSGs examined, indicating that H. pylori can enhance the functional ability of EBV to drive oncogenesis but is limited in its own capacity to drive the oncogenic phenotype of gastric epithelial cells (Fig. S6B and C).

## DISCUSSION

Oncogenesis associated with approximately 20% of human cancers is driven by infectious agents as the causative inducers (24). Epigenetic modulators alter the expression of cellular genes, which modifies the microenvironment to one suitable for oncogenesis. EBV is known to promote oncogenesis through epigenetic modifications resulting in transcription reprogramming of infected cells. These changes contribute to the development of cancers of epithelial and lymphoid origin (25, 26).

In the present study, NCI-N87 gastric epithelial cells were used; the properties of those cells closely mimic the properties of primary gastric epithelial cells based on the cell-cell interaction mechanisms, analysis of adherent junction, and expression of cytoskeleton and cell-matrix proteins (14, 15). Notably, H. pylori specifically targets and resides at the adherent junction (27, 28). This prompted us to examine a preferable model by which EBV infection can successfully occur in H. pylori-exposed NCI-N87 gastric cells. Forty-eight hours of preincubation with H. pylori resulted in a consistently higher EBV DNA copy number as well as in higher GFP expression with GFP-EBV–H. pylori coinfection. Earlier studies showed that H. pylori can modulate expression of a number of cellular genes associated with inflammation. Furthermore, this infection system provided a suitable niche for EBV-driven proliferation (29, 30). Our studies demonstrated that exposure of NCI-N87 to H. pylori prior to EBV infection significantly increased the efficiency of EBV infection and led to increased viral genome copy numbers in the gastric epithelial cells. To determine if this was unique to NCI-N87 cells, we replicated the coinfection experiment in AGS cells (representing another gastric epithelial cell line), which showed similar effects (data not shown). These results support our hypothesis that exposure to H. pylori can enhance the inflammatory response which facilitates signaling activities and drives EBV-mediated proliferation of gastric epithelial cells. Further, to address the possibility of passive GFP uptake or enhancement of fluorescence caused by dead cellular aggregates, we performed a passive GFP uptake assay and a time course analysis post-EBV infection. The results further validated the findings revealed by an increase in the level of GFP expression and in viral copy numbers and indicated that the increase in the GFP signal can be caused only by infection and virus replication. The HEK293T cells expressing GFP alone were treated under experimental conditions similar to those used in the EBV preparation, and culture supernatants were used for incubation with the NCI-N87 cells. The results showed that neither dead cells nor cellular debris contributed to the GFP fluorescence observed in our experimental system.

Latent and lytic genes (EBNA1 and BZLF1 genes, respectively) displayed higher expression than the control at increasing time points in our quantitative PCR and immunofluorescence analyses. This trend for EBNA1 and BZLF1 expression in coinfections performed with H. pylori suggests that H. pylori contributed to the increased efficiency of EBV infection and to early replication. This increase in GFP expression was most likely a direct consequence of increased levels of infection and viral replication leading to increased persistent production of virus particles during the early period of infection.

Furthermore, using H. pylori cell-free culture supernatant, we were able to replicate the results described above. This provided strong evidence that the enhanced levels of infectivity and viral copy numbers were due to an H. pylori-encoded secretory factor. The higher expression of latent as well as lytic genes carried by EBV and the infection of a fresh culture of gastric epithelial cells with progeny virus suggested that the results were the consequence of increased EBV replication in the cells in the presence of H. pylori. Also, H. pylori coinfection resulted in a drop in viral copy numbers, although the drop was less than that seen with EBV alone in the presence of acyclovir (ACV), which inhibits herpesvirus replication at inhibitory concentrations (31). It should be noted that the presence of H. pylori can alter the microenvironment of the gastric epithelial cells and may enhance the efficiency of viral infection (32).

The cytotoxin-associated gene A (CagA) protein is a secretory antigen located on the surface of H. pylori secretory vesicles (32). It forms a complex with cell junction proteins, causing a morphological dysplastic alteration in epithelial cells (27). This alteration is important and contributes to the oncogenic process (33). Furthermore, CagA-positive H. pylori is associated with aggressive forms of disease, such as a severe form of gastritis, peptic ulcers, and GC (34–36). H. pylori CagA-positive strains can also alter the inflammation-driven cellular response and show increased levels of interleukin-8 (IL-8) expression (35, 37). Our results support the hypothesis that human gastric epithelial cells have enhanced susceptibility to EBV infection in the presence of the H. pylori wild-type strain (carrying the CagA gene). An H. pylori CagA mutant showed a significant reduction in the level of viral infection similar to that seen with EBV alone, suggesting that CagA was a major contributor to the increased efficiency of infection. Similar observations were made with cell-free culture supernatants of the wild-type strain and the CagA-deleted mutant, indicating that the active factor was secreted in the culture medium. Cells have more than one mechanism for CagA uptake by gastric cells. The first mechanism is that of utilizing the type IV bacterial secretion system that directly injects the CagA factor into the cells (38). Alternatively, binding of CagA to cell surface lipid phosphatidyl serine activated integrins, and rearrangement of actin leads to endocytosis of CagA or its fragments and causes phenotypic alteration in gastric epithelial cells (39). Here, our results supported the hypothesis that the alternative (second) mechanism is the one used, as the culture supernatant showed enhanced levels of EBV infection and copy numbers.

Small-molecule effectors encoded by microbial agents can drive oncogenesis. Additionally, interspecies competition and synergies between resident microbes can have a significant impact on functional outcomes. In particular, as we have observed in the context of H. pylori and EBV dynamics in GC, phosphorylated CagA interacts with the proto-oncogene SPH1, a host tyrosine phosphatase, and this, in turn, dampens the CagA-induced oncogenic action (38). Conversely, EBV infection leads to promoter hypermethylation of SPH1 which rescues the phosphorylated CagA from phosphatase and enhances gastric oncogenesis (38). Our results add to those results and show that TSGs are also regulated and that CagA is an important H. pylori*-*encoded factor.

Aberrant DNA methylation in GC is an important driver of the oncogenic process and is associated with both H. pylori infection and EBV infection (40–42). Three major classes of DNMTs, namely, DNMT class 1 (DNMT1) (involved in postreplication maintenance of the existing methylation pattern of newly synthesized strands) and DNMT3A and DNMT3B (DNMT3A/3B) (involved in the *de novo* methylation of CpGs), are important contributors to cellular and viral methylation patterns (43). Another member of the DNMT3 family, DNMT3L, is catalytically inactive but has a role in the development of germ cells (44). However, binding of DNMT3L with DNMT3A/3B increases their catalytic activity by as much as 15-fold (44). The role of DNMT2 is not completely understood; DNMT2 is possibly associated with methylation of tRNAs and has a protective function (45, 46). Empirical evidence indicates that the DNMTs encoded by the host cells, and not the H. pylori-encoded DNMTs, contribute to DNA methylation of the host genome (47). Further, more-exhaustive analysis failed to detect any H. pylori-encoded DNMTs in the H. pylori secretory proteome (32). Here, we show that coinfection by H. pylori and EBV induced expression of the epigenetic effectors of the host cell, including DNMTs. Further, significant upregulation of DNMT3A/3B led to methylation of CpG islands of TSGs in the host genome. Notably, EBV is a known epigenetic modulator regulating a range of cellular processes important for oncogenesis (19). In this coinfection model system, we observed a drastic shift in the host epigenome. There are several high-throughput methods for methylation detection, such as bisulfite sequencing, pyrosequencing, and next-generation sequencing (48–50). However, we elected to use an EpiTect methyl II PCR array system based on the following criteria. First, it is pathway focused and rapid; second, it is reliable, reproducible, and cost-effective. Most importantly, its outcome is comparable to those obtained using other techniques, including bisulfite sequencing (51).

Upregulation of DNMTs is directly responsible for promoter hypermethylation of a broad range of tumor suppressor genes, which in turn results in decreased expression of TSGs (20). Further, in our current study, we showed that exposure of gastric epithelial cells to H. pylori prior to EBV infection led to promoter hypermethylation of TSGs and to downregulation of their transcription. This led to increased cell proliferation and an enhanced oncogenic phenotype as seen in our colony formation assays. These results support those of a previous study which showed that a number of TSGs had enhanced methylation patterns in gastric cells (52).

Multiple pathways and cellular processes are affected by extensive promoter methylation of TSGs. Cyclin-dependent kinase inhibitor 2A blocks MDM2-mediated degradation of p53 (53). CDKN2A interacts with MDM2 and inhibits the cytoplasm-nuclear movement of MDM2 (53). This stabilizes p53 and enhances p53-mediated apoptosis (53). CDKN2A also binds to E2F1 and Bcl6 and suppresses their transcription activity (54). Downstream targets of BCL6 are proteins involved in DNA damage sensing and cell cycle proliferation (55). CDKN1A (p21), however, was not shut down, which highlights its importance in the context of EBV-mediated oncogenesis (56). MGMT (methyl guanine DNA methyl transferase) is associated with several cancer types, including colorectal cancer, lung cancer, and glioblastoma (57). MGMT is involved in the DNA repair process, protecting against the activity of alkylating agents, and caused the alkylated guanidine to revert to its natural state by transferring an alkyl group to specific cysteine residues of MGMT (57). The APC (adenomatous polyposis coli) promoter is also known to be methylated in aggressive forms of breast cancer and other cancers (58) and serves as an inhibitor of Wnt signaling (59).

Loss of the phosphatase and tensin homologue (PTEN) is associated with many cancers and functions as a negative regulator of the PI3-kinase/AKT pathway by downregulating PIP3 (phosphatidyl inositol 3,4,5-triphosphate) levels (60). In one study on EBV-positive GC, PTEN was found to be hypermethylated (61). Upon infection with recombinant EBV, GC cell lines MKN1 and MKN7 showed DNMT1 upregulation along with hypermethylation of the regulatory region of the PTEN gene (61). This demonstrates that the association of infection, DNMT-mediated changes in methylation of the cellular genome, and dysregulation of cellular signaling was important for oncogenesis (61). Another study showed that epigenetic alterations in EBV-positive GC downregulated meiotic recombination protein Rec8 in 100% of the cases examined, thereby causing loss of its tumor-suppressive effect (62). This represents a direct mechanism by which pathogen-driven epigenetic alteration is linked to the loss of tumor suppressor function, causing tumorigenic progression (Fig. 5).

The present study demonstrated an epigenetic mechanism which specifically involved DNMT-mediated TSG silencing in infected gastric epithelial cells. It establishes the H. pylori-EBV nexus, which creates a tumor microenvironment suitable for gastric epithelial cell proliferation and oncogenesis. Thus, H. pylori exposure aids in proliferation of EBV-infected gastric epithelial cells through modulation of the cell cycle, apoptosis, and DNA damage repair pathways, leading to uncontrolled proliferation of virus-infected cells and development of gastric cancer.

## MATERIALS AND METHODS

### Ethics statement.

All the work was performed in accordance with ethical guidelines of University of Pennsylvania and Helsinki recommendations.

### Cells, virus, and bacterial cultures.

The NCI-N87 and AGS human gastric epithelial cell lines were obtained from the American Type Culture Collection (ATCC); the cell lines are negative for EBV (62). As described earlier, HEK293T cells containing GFP-EBV were cultured and used for EBV amplification (63).

### Induction of virus from HEK293T cells containing GFP-EBV.

Cultured 293T-GFP-EBV cells were induced for 5 days with 20 ng/ml tetradecanoyl phorbol acetate (TPA) and 3 mM butyric acid (Sigma-Aldrich Corp., St. Louis, MO), and the virus was purified as described previously (64).

### Infection of NCI-N87 and AGS cells with GFP-EBV and H. pylori*.*

NCI-N87 cells were grown over poly-l-lysine-coated sterile coverslips in 6-well plates. When the cells reached 60% confluence, H. pylori exposure was set up using a 0.4-µM-pore-size transwell. After 48 h, the virus was added to the culture medium.

A primary H. pylori culture was washed twice with sterile 1× phosphate-buffered saline (PBS) and suspended in serum- and antibiotic-free RPMI medium. Infection was performed in a 6-well plate using a transwell system (Corning Inc., Lowell, MA) (0.4 µM pore size, 24-mm diameter). NCI-N87 and AGS cells were incubated with H. pylori for 48 h prior to the GFP-EBV treatment.

### DNA and RNA extraction.

DNA was isolated using a DNeasy blood and tissue kit (Qiagen, Valencia, CA) following the manufacturer’s instructions. Extracellular viral DNA was extracted from culture supernatants as previously described (65), and DNA was quantified using real-time quantitative PCR (RT-qPCR) performed with previously described primers (20, 63).

### Replication inhibition by ACV.

Acyclovir [ACV; 9-(2-hydroxyethoxy methyl) guanine] is an antiviral. A 25 µM concentration of ACV was used for significant and reproducible EBV replication inhibition in NCI-N87 cells (63).

### Profiling of DNA methylation.

DNA samples obtained after infection were analyzed using human tumor suppressor gene EpiTect DNA methylation PCR array signature panel EAHS551ZC (SABiosciences, Frederick, MD) following the manufacturer’s instructions. Please refer to Text S1 in the supplemental material for a detailed description of the procedure.

10.1128/mBio.00649-18.1 TEXT S1  Supplemental methods. Download TEXT S1, DOCX file, 0.02 MB. Copyright © 2018 Pandey et al. 2018 Pandey et al. This is an open-access article distributed under the terms of the Creative Commons Attribution 4.0 International license.

### Real-time quantitative PCR.

Real-time quantitative PCR (RT-qPCR) analysis was carried out using previously described methods (66). Details are provided in Text S1 and elsewhere in Materials and Methods.

### Cell proliferation assay.

A cell proliferation assay was performed using trypan blue exclusion and crystal violet staining methods as previously described (67, 68). Brief descriptions are provided in Text S1 and elsewhere in Materials and Methods.

### Statistical analysis.

Data were statistically analyzed using Student’s *t* test. All the results were derived from triplicate experiments (***, *P* value of <0.001 [using Student’s *t* test and Graph Pad Prizm version 6]).

### Data availability.

All of the data sets that support the findings of this study are available in the present manuscript in either the main text or the supplemental material.
