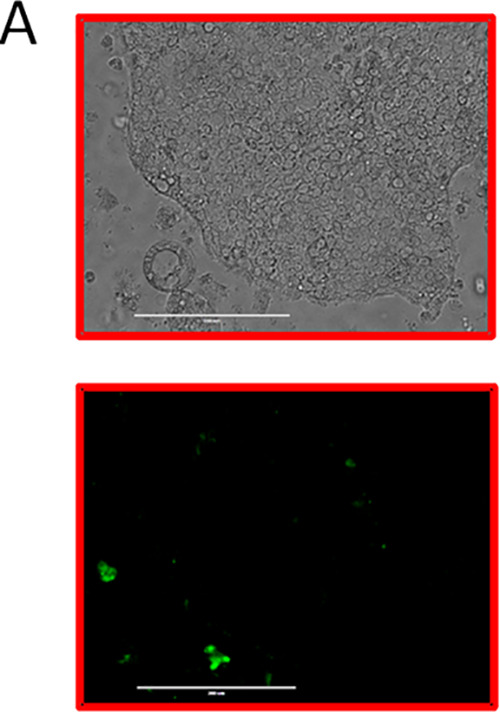# Erratum for Pandey et al., “Epigenetic Regulation of Tumor Suppressors by Helicobacter pylori Enhances EBV-Induced Proliferation of Gastric Epithelial Cells”

**DOI:** 10.1128/mBio.03074-20

**Published:** 2020-12-15

**Authors:** Saurabh Pandey, Hem Chandra Jha, Sanket Kumar Shukla, Meghan K. Shirley, Erle S. Robertson

**Affiliations:** a Departments of Otorhinolaryngology-Head and Neck Surgery, and Microbiology, the Tumor Virology Program, Abramson Cancer Center, Perelman School of Medicine at the University of Pennsylvania, Philadelphia, Pennsylvania, USA

## ERRATUM

Volume 9, no. 2, e00649-18, 2018, https://doi.org/10.1128/mBio.00649-18. In Fig. 4A, there was a duplicated image in the bright-field and GFP entries for the CagA mutant strain at 1 dpi. There are no corresponding changes to the text or figure legend.

**Figure:**