# Supplementary figures and images for: Epigenetic Regulation of Tumor Suppressors by Helicobacter pylori Enhances EBV-Induced Proliferation of Gastric Epithelial Cells
Source: mBio. 2018 Apr 24;9(2):e00649-18. doi: 10.1128/mBio.00649-18 (PMC5915740; doi:10.1128/mBio.00649-18)

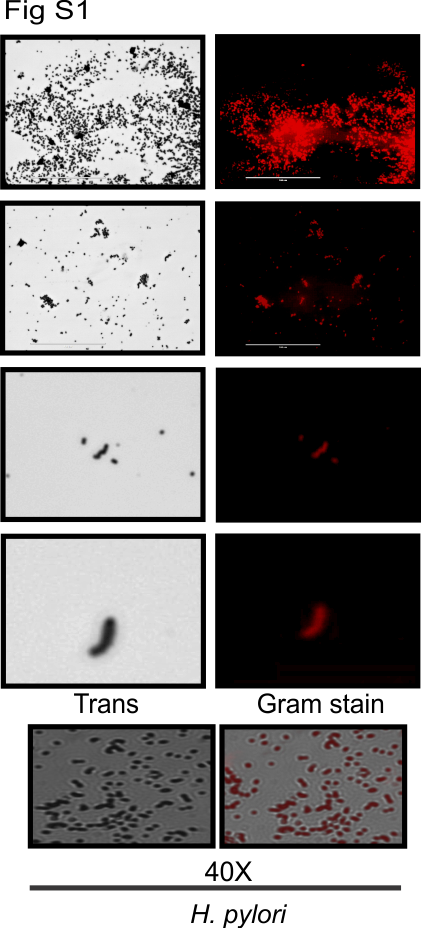

Supplement: FIG S1 [file mbo002183857sf1.tif]

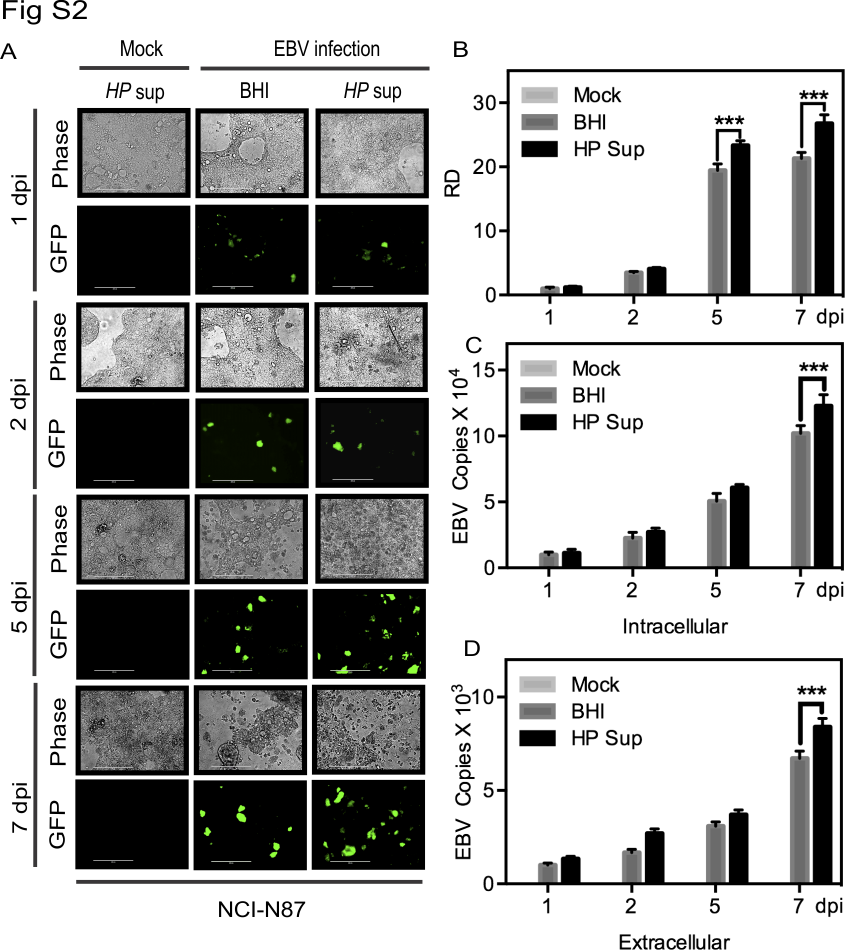

Supplement: FIG S2 [file mbo002183857sf2.tif]

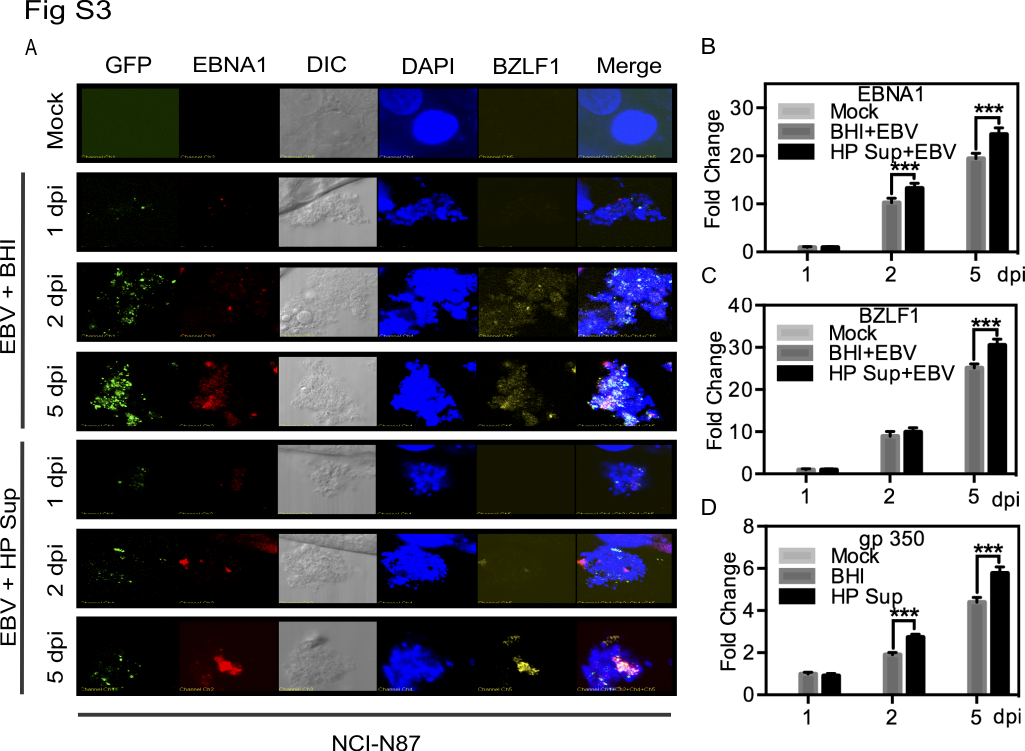

Supplement: FIG S3 [file mbo002183857sf3.tif]

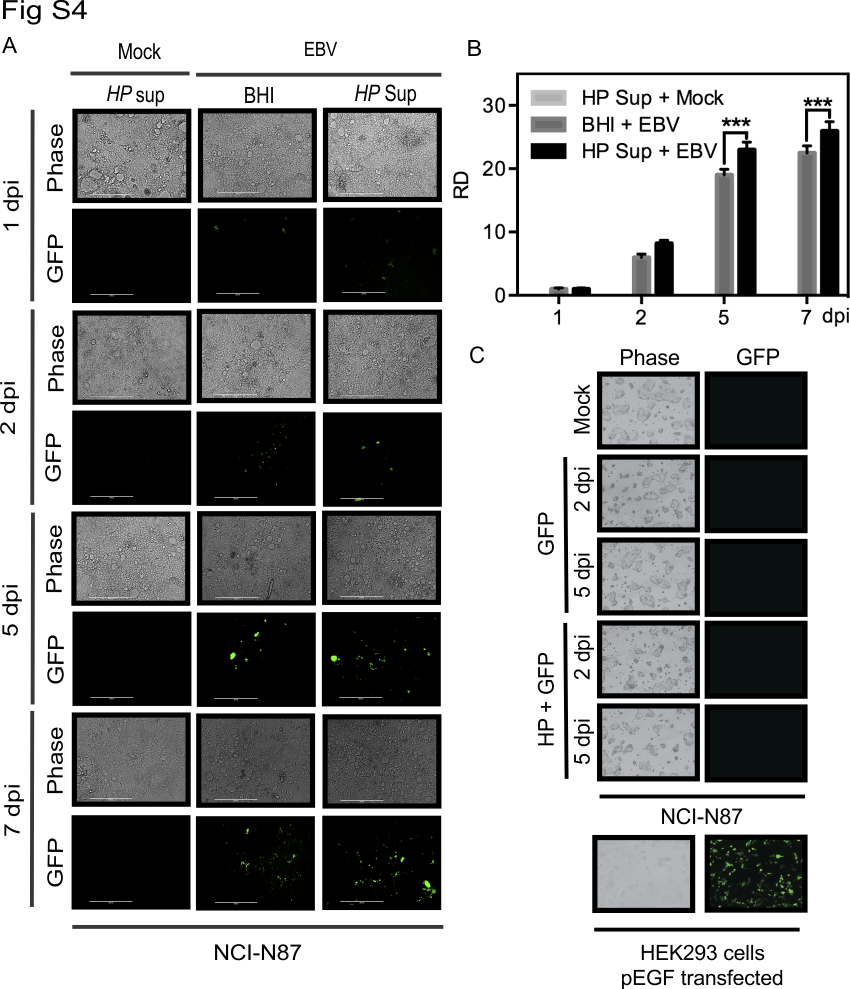

Supplement: FIG S4 [file mbo002183857sf4.tif]

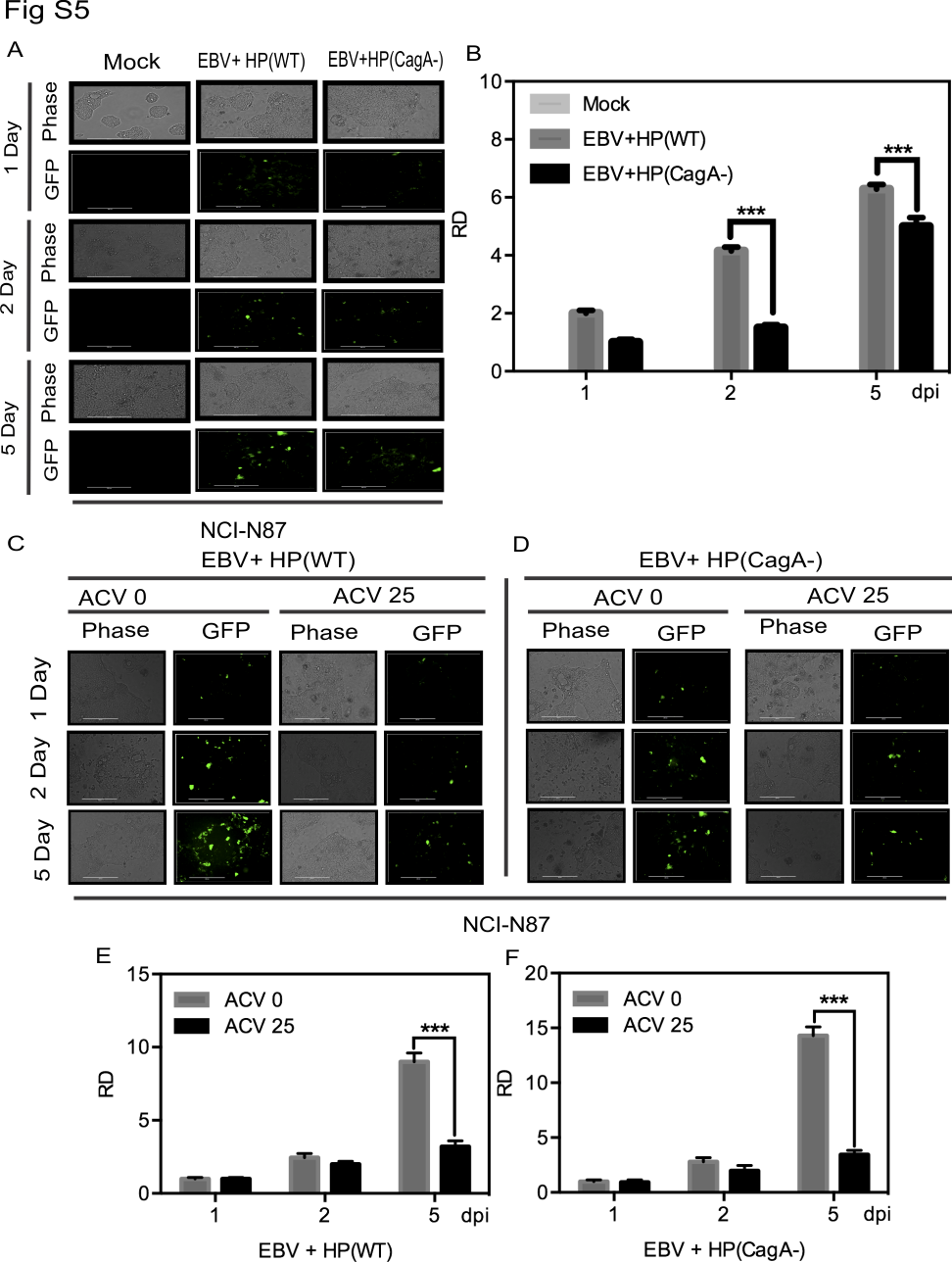

Supplement: FIG S5 [file mbo002183857sf5.tif]

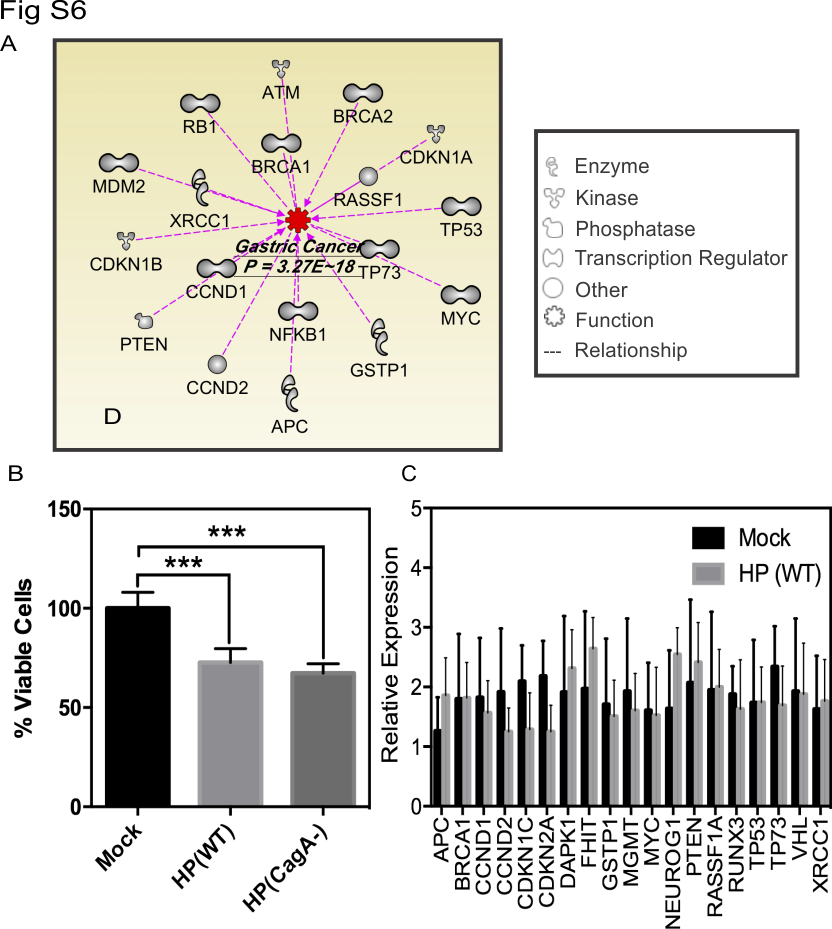

Supplement: FIG S6 [file mbo002183857sf6.tif]
