# Supplementary material for: Epigenetic Regulation of Tumor Suppressors by Helicobacter pylori Enhances EBV-Induced Proliferation of Gastric Epithelial Cells
Source: mBio. 2018 Apr 24;9(2):e00649-18. doi: 10.1128/mBio.00649-18 (PMC5915740; doi:10.1128/mBio.00649-18)
Supplement: TABLE S1 [file mbo002183857st1.docx]

**Table S1. List of TSGs in methylation profiling**

| S. No. | Gene | RefSeq | Description |
| --- | --- | --- | --- |
| 1 | APC | NM_000038 | Adenomatous polyposis coli |
| 2 | BRCA1 | NM_007294 | Breast cancer1, early onset |
| 3 | CDH1 | NM_004360 | Cadherin1 or E-cadherin, (epithelial) |
| 4 | CHD13 | NM_001257 | Cadherin13 or H-cadherin, (heart) |
| 5 | CDKN2A | NM_000077 | Cyclin–dependent kinase inhibitor 2A (P16, inhibits CDK4) |
| 6 | DAPK1 | NM_004938 | Death associated protein kinase 1 |
| 7 | ESR1 | NM_000125 | Estrogen receptor1 |
| 8 | FHIT | NM_002012 | Fragile histidine triad gene |
| 9 | GSTP1 | NM_000852 | Glutathione S-transferase pi 1 |
| 10 | MGMT | NM_002412 | O-6-methylguanine-DNA methyltransferase |
| 11 | MLH1 | NM_000249 | MutL homolog 1, colon cancer, non polyposis type 2(E. coli) |
| 12 | NEUROG1 | NM_006161 | Neurogenin1 |
| 13 | PDLIM4 | NM_003687 | PDZ and LIM domain 4 |
| 14 | PTEN | NM_000314 | Phosphatase and tensin homolog |
| 15 | RARB | NM_000965 | Retinoic acid receptor β |
| 16 | RASSF1 | NM_007182 | Ras associated domain family member 1 |
| 17 | RUNX3 | NM_004350 | Runt related transcription factor 3 |
| 18 | SOCS1 | NM_003745 | Suppressor of cytokine signaling 1 |
| 19 | TIMP3 | NM_000362 | TIMP metallopeptidase inhibitor 3 |
| 20 | TP73 | NM_005427 | Tumor protein p73 |
| 21 | VHL | NM_000551 | Von Hippel-Lindau tumor suppressor |
| 22 | WIFI | NM_007191 | WNT inhibitory factor 1 |
