# Supplementary material for: Epigenetic Regulation of Tumor Suppressors by Helicobacter pylori Enhances EBV-Induced Proliferation of Gastric Epithelial Cells
Source: mBio. 2018 Apr 24;9(2):e00649-18. doi: 10.1128/mBio.00649-18 (PMC5915740; doi:10.1128/mBio.00649-18)
Supplement: TABLE S2 [file mbo002183857st2.docx]

**Table S2. List of categories of TSGs in methyl profiling***

| S. No. | Category | TSGs |
| --- | --- | --- |
| 1 | Apoptosis | BRCA1, CDKN2A, DAPK1, GSTP1, MGMT (AGT), PTEN, RUNX3, TP73, VHL |
| 2 | Cell Adhesion | APC, CDKN2A |
| 3 | Cell Cycle | APC, BRCA1, FHIT, NEUROG1, PTEN, RASSF1, RUNX3, TP73, VHL |
| 4 | DNA Damage Repair | APC, BRCA1, MGMT (AGT), TP73 |
| 5 | Signal Transduction | APC, BRCA1, PTEN, RASSF1, SOCS1 |
| 6 | Transcription Factors | BRCA1, NEUROG1, RUNX3, TP73, VHL |

*****http://www.sabiosciences.com/dna_methylation_product/HTML/EAHS-551Z.html
