# Supplementary material for: Epigenetic Regulation of Tumor Suppressors by Helicobacter pylori Enhances EBV-Induced Proliferation of Gastric Epithelial Cells
Source: mBio. 2018 Apr 24;9(2):e00649-18. doi: 10.1128/mBio.00649-18 (PMC5915740; doi:10.1128/mBio.00649-18)
Supplement: TABLE S3 [file mbo002183857st3.docx]

**TABLE S3: List of categories of TSGs regulated by transcription reprogramming**

| S. No. | Category | TSGs |
| --- | --- | --- |
| 1 | Apoptosis | BRCA1, CDKN1A, CDKN1B, E2F1, GSTP1, MDM2, MGMT NFKB1, PTEN, TP53, TP73, VHL |
| 2 | Cell Adhesion | APC, CDKN2A |
| 3 | Cell Cycle | APC, ATM, BRCA1, BRCA2, CCND1, CCND2, CDKN1A, CDKN1B, E2F1, MDM2, MYC, PTEN, RASSF1, RB1, TGFB1, TP73, VHL |
| 4 | DNA Damage Repair | APC, ATM, BRCA1, BRCA2, CDKN1A, E2F1, MGMT, TP53, TP73, XRCC1 |
