# Supplementary material for: Epigenetic Regulation of Tumor Suppressors by Helicobacter pylori Enhances EBV-Induced Proliferation of Gastric Epithelial Cells
Source: mBio. 2018 Apr 24;9(2):e00649-18. doi: 10.1128/mBio.00649-18 (PMC5915740; doi:10.1128/mBio.00649-18)
Supplement: TEXT S1 [file mbo002183857s1.docx]

**Supplementary Information**

**Material and Method:**

**Cell culture:**

NCI-N87 and AGS cells were cultured in 100 mm dishes in RPMI 1640 supplemented with 10 FBS, 2mM L-glutamine and 50 units of penicillin as recommended by ATCC.

***HP* culture, quantitation and infection:**

Strains of *HP* were cultured using Compy Container System with GasPak EZ. Liquid culture was maintained in brain heart infusion media (Difco Laboratories, Detroit, Mich.) supplemented with 10% FBS. In the solid medium, *HP* was cultured on Blood agar plate (Remel, Thermo Fisher Scientific Blood Agar Plate R01200). Cultured *HP* 60190 was also stained by gram staining method. *HP* was quantitated making a standard curve with OD_550_ and colony forming Units after 1:1000 dilutions. Infection was set up at 5 multiplicity of infection (MOI) using transwell system after washing cells with PBS and diluting in serum free-antibiotic free RPMI.

**Calculation of MOI:** 10-fold serial dilution of *HP* culture, 1:10^2^ to 1:10^5^ was prepared in the culture media. Absorbance was measured at 550nm using media as a blank and dilution were plated in blood agar plate for enumerating CFU after 4-5 days. Growth curve was prepared and using that, absorbance vs CFU/ml formula was calculated (e.g. 0.1 OD550 ≈10^7^ CFU/ml). Based on number of cells to be infected and multiplicity of infection to be used required dilutions and volume was calculated.

For infection with *HP* culture medium, we quantitated the presence of *HP* in the culture, and the fraction of cell-free culture medium corresponding to the required MOI was used.

**EBV Preparation, Quantitation and Infection:**

293T-GFP-EBV cells were cultured and induced with TPA (20ng/ml) and BA (3 mM) for 5 days. Virus particles were harvested by centrifugation at 3000rpm for 30 minutes, and then filtered through a 0.45μM membrane filter. Filtrate was concentrated by ultracentrifugation at 23,500 rpm at 4^o^C. Purified virus is dissolved in 1 ml of complete media. Virus DNA copy number was determined by qPCR using Namalwa cells having 2 copy number of EBV DNA (70, 71). To set-up infection, cells were incubated with 293T-GFP-EBV supernatant with MOI of 5 in 1 ml.

**Profiling of DNA Methylation:**

DNA samples after obtained after infection were analyzed using the Human Tumor Suppressor Genes DNA Methylation PCR Array signature panel EAHS551ZC (SABiosciences, Frederick, MD, USA) following the manufacturer’s instructions and does not need bisulfite sequencing to determine methylation of CpG islands at regulatory regions.

In brief, the technique relies on the quantitative estimation of promoter DNA methylation by RT-PCR where genomic DNA of samples was isolated and digested by the set of methylation-sensitive and dependent restriction enzymes along with undigested and double digested control reactions. Afterward, samples were amplified by RT-PCR in pre-coated target gene promoter based 96-well plate system and cycle threshold (Ct) values were obtained. Ct was used to calculate the percentage of DNA methylation at a given restriction site (72). The analysis was performed by an algorithm provided by the manufacturer (http://www.sabiosciences.com/dna_methylation_data_analysis.php). The algorithm has quality control criteria to include or omit individual gene-related data. The data was displayed as a heat map after normalization with mock infection for individual gene.

**Real-time quantitative** **PCR:**

Real-time quantitative PCR (RT-qPCR) analysis was carried out using previously described methods (67). In short, cells were harvested by using Trizol reagent (Invitrogen Inc., Carlsbad, CA), and the total RNA isolated. The RNA was treated with DNase I (Invitrogen Inc., Carlsbad, CA). The RNA was further used for the synthesis of cDNA using a reverse transcription kit of Applied Biosystems Inc. (Foster City, CA) following manufacturer’s instructions. Real-time PCR analysis was performed using power SYBR master mix on a step one plus PCR system (Applied Biosystems Inc, foster city, CA). All experiments were performed in triplicate; the relative quantitation values were calculated by the 2-delta-delta Ct method and normalized using 18S rRNA control. Amplified products were analyzed by melting curve analysis to verify the specificity of the amplicons.

**Cell Proliferation assay:**

NCI-N87 cells were cultured in 6-well plates and subjected to co-infection using 0.4 μm transwell system. At different time points, viable cells were counted using trypan blue exclusion assay (68). Repeating, the plates were washed twice with PBS, fixed with 4% paraformaldehyde and stained with 0.1% crystal violet dye. After washing with PBS to remove the non-specific dye, the plates were dried and scanned using a Bio-Rad ChemiDoc system (Bio-Rad Laboratories, Hercules, CA) (69)

**Supplementary References**

67. Saha A, Murakami M, Kumar P, Bajaj B, Sims K, Robertson ES. 2009. Epstein-Barr virus nuclear antigen 3C augments Mdm2-mediated p53 ubiquitination and degradation by deubiquitinating Mdm2. J Virol 83:4652–69.

68. Banerjee S, Lu J, Cai Q, Sun Z, Jha HC, Robertson ES. 2014. EBNA3C Augments Pim-1 Mediated Phosphorylation and Degradation of p21 to Promote B-Cell Proliferation. PLoS Pathog 10.

69. Jha HC, Sun Z, Upadhyay SK, El-Naccache DW, Singh RK, Sahu SK, Robertson ES. 2016. KSHV-Mediated Regulation of Par3 and SNAIL Contributes to B-Cell Proliferation. PLoS Pathog 12:1–31.

70. Henderson A, Ripley S, Heller M, Kieff E. 1983. Chromosome site for Epstein-Barr virus DNA in a Burkitt tumor cell line and in lymphocytes growth-transformed in vitro. Proc Natl Acad Sci U S A 80:1987–1991.

71. Matsuo T, Heller M, Petti L, O’Shiro E, Kieff E. 1984. Persistence of the entire Epstein-Barr virus genome integrated into human lymphocyte DNA. Science 226:1322–1325.

72. Oakes CC, Salle S La, Trasler JM, Robaire B. 2009. DNA Methylation:Restriction Digestion and Real-Time PCR (qAMP), p. 271–280. *In* DNA Methylation: Methods and Protocols.
